# Spermine oxidase promotes *Helicobacter pylori*-mediated gastric carcinogenesis through acrolein production

**DOI:** 10.1038/s41388-024-03218-7

**Published:** 2024-11-10

**Authors:** Kara M. McNamara, Johanna C. Sierra, Yvonne L. Latour, Caroline V. Hawkins, Mohammad Asim, Kamery J. Williams, Daniel P. Barry, Margaret M. Allaman, Irene Zagol-Ikapitte, Paula B. Luis, Claus Schneider, Alberto G. Delgado, M. Blanca Piazuelo, Regina N. Tyree, Kate S. Carson, Yash A. Choksi, Lori A. Coburn, Alain P. Gobert, Keith T. Wilson

**Affiliations:** 1 Division of Gastroenterology, Hepatology, and Nutrition, Department of Medicine, Vanderbilt University Medical Center, Nashville, TN 37232 USA; 2 Program in Cancer Biology, Vanderbilt University School of Medicine, Nashville, TN 37232 USA; 3 Center for Mucosal Inflammation and Cancer, Vanderbilt University School of Medicine, Nashville, TN 37232 USA; 4 Department of Pathology, Microbiology, and Immunology, Vanderbilt University School of Medicine, Nashville, TN 37232 USA; 5 Warren Center for Neuroscience Drug Discovery, Vanderbilt University, Franklin, TN 37067 USA; 6 Department of Pharmacology, Vanderbilt University School of Medicine, Nashville, TN 37232 USA; 7 Veterans Affairs Tennessee Valley Healthcare System, Nashville, TN USA

**Keywords:** Gastric cancer, Oncogenes

## Abstract

*Helicobacter pylori* is the primary cause of gastric cancer, and there is a need to discover new molecular targets for therapeutic intervention in *H. pylori* disease progression. We have previously shown that spermine oxidase (SMOX), the enzyme that catabolizes the back-conversion of the polyamine spermine to spermidine, is upregulated during infection and is associated with increased cancer risk in humans. We sought to determine the direct role of SMOX in gastric carcinogenesis during *H. pylori* infection. In this study, we demonstrate that transgenic FVB/N insulin-gastrin (INS-GAS) mice that develop gastric carcinoma with *H. pylori* infection were protected from cancer development with *Smox* deletion. RNA sequencing revealed that genes associated with the immune system and cancer were downregulated in the infected *Smox*^*–/–*^ mice. Furthermore, there was a decrease in cell proliferation and DNA damage in infected *Smox*^*–/–*^ animals. There was significant generation of adducts of the highly reactive electrophile acrolein, a byproduct of SMOX activity, in gastric tissues from *H. pylori*-infected humans and wild-type, but not *Smox*^*–/–*^ mice. Genetic deletion of *Smox* in murine organoids or chemical inhibition of SMOX in human gastric epithelial cells significantly reduced generation of acrolein induced by *H. pylori*. Additionally, acrolein-induced DNA damage in gastric epithelial cells was ablated with the electrophile scavenger 2-hydroxybenzylamine (2-HOBA). Gastric acrolein adduct levels were attenuated in infected INS-GAS mice treated with 2-HOBA, which exhibit reduced gastric carcinoma. These findings implicate SMOX and acrolein in *H. pylori*-induced carcinogenesis, thus indicating their potential as therapeutic targets.

## Introduction

Gastric cancer is the fifth most common diagnosed cancer and is currently the fifth leading cause of cancer mortality worldwide [1]. Infection with *Helicobacter pylori* is associated with 89% of non-cardia gastric cancers and is therefore the strongest known risk factor for developing gastric cancer [2]. Although approximately 4.4 billion people are infected with *H. pylori*, making it the most common chronic bacterial infection worldwide [3], only a subgroup of infected individuals progress through the histological steps of the “Correa Cascade” from chronic inflammation to atrophic gastritis, intestinal metaplasia, dysplasia, and ultimately gastric adenocarcinoma [4, 5].

We have previously shown that polyamines play various functions in the pathophysiology of *H. pylori* infection. The catabolism of L-ornithine by ornithine decarboxylase generates the polyamine putrescine, which is sequentially metabolized to spermidine and spermine by spermidine synthase and spermine synthase, respectively [6, 7]. Spermine is back-converted to spermidine by the enzyme spermine oxidase (SMOX), generating the byproducts H_2_O_2_ and 3-aminopropanol [8]. We have previously demonstrated the induction of SMOX in myeloid cells and gastric epithelial cells (GECs) during *H. pylori* infection in humans and rodents [9–13]. In macrophages, the activity of SMOX supports apoptosis and dampens antimicrobial activity [9, 10], thus participating in the immune escape of *H. pylori*. In addition, SMOX contributes to DNA damage and apoptosis in GECs [11–13] and a correlation between SMOX expression and disease progression has been reported in *H. pylori*-infected patients [13]. However, the direct proof that SMOX plays a role in *H. pylori*-mediated carcinogenesis has not been demonstrated.

While the pathophysiological role of SMOX-derived H_2_O_2_ has been thoroughly studied, that of 3-aminopropanol, and its spontaneous and immediate conversion to acrolein, a highly reactive electrophile, has yet to be elucidated. Acrolein is endogenously produced through 1) polyamine metabolism, 2) degradation of threonine by myeloperoxidase, 3) metabolism of cancer drugs, and 4) lipid peroxidation of polyunsaturated fatty acids [14]. It can also be consumed through the diet, including from fried foods and alcoholic beverages, and exogenously through cigarette smoke and automobile exhaust [14]. Acrolein can react with deoxyguanosine in DNA and nucleophilic residues, i.e. cysteine, histidine, and lysine, in proteins to form adducts, which can lead to mutagenesis and disruption of gene expression [14]. Due to its ability to induce DNA damage and inhibit DNA repair [15], acrolein has been implicated in cancers associated with tobacco smoking including lung [15], oral [16], and bladder [17]. Additionally, acrolein was shown to induce DNA damage in colorectal tumor tissues and promote colon tumorigenesis through the activation of the RAS/MAPK signaling pathway [18].

In this study, we examine *Smox*-deficiency in an established gastric cancer mouse model, FVB/N insulin-gastrin (INS-GAS) mice, mediated by *H. pylori* infection. We present evidence that the genetic ablation of *Smox* results in decreased progression to gastric cancer, suppression of proinflammatory, chemokine, T-cell associated, and carcinogenic genes, and reduced cell proliferation and DNA damage during *H. pylori* infection. We show that SMOX supports acrolein generation in *H. pylori*-infected mice, in murine and human gastric-derived organoids, and in human GECs. Moreover, acrolein production mediates DNA damage in human GECs, which is decreased by the electrophile scavenger 2-hydroxybenzylamine (2-HOBA) [19]. Furthermore, we provide evidence that acrolein adducts are increased in patients with *H. pylori* gastritis or low-grade dysplasia (LGD), and in infected INS-GAS mice. In the latter, the adducts are reduced by *Smox* deletion or 2-HOBA treatment. Thus, our data indicate that SMOX activity affects the progression from gastric dysplasia to cancer and implicates acrolein in gastric carcinogenesis by enhancing DNA damage.

## Results

### Reduction of *H. pylori*-induced carcinogenesis in INS-GAS mice with *Smox* deletion

**Fig. 1:**
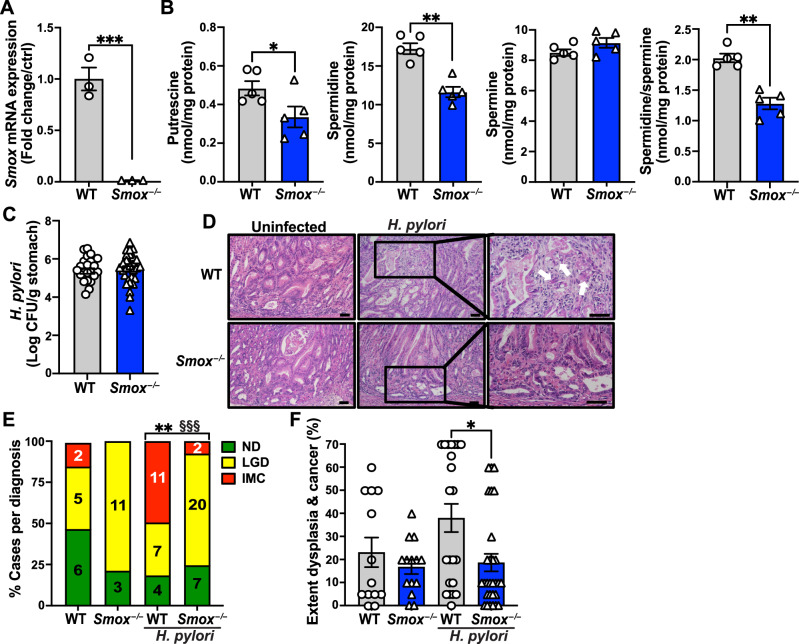
Effect of *Smox* deletion on *H. pylori* pathogenesis in a gastric cancer model. *Smox* mRNA expression (**A**; *n* = 3 per genotype) and polyamine concentrations (**B**; *n* = 5 per genotype) were determined in the gastric tissues of naïve WT and *Smox*^*–/–*^ animals by RT-real-time PCR and mass spectrometry, respectively. Then, animals were infected or not with *H. pylori* PMSS1 for 8 weeks. *H. pylori* colonization was determined by serial dilution and culture (**C**). Tissues stained by H&E (**D**) were used to analyze the frequency of dysplasia and cancer (**E**) and their extent (**F**); ND no dysplasia; in **D**, scale bars represent 100 μm (left and center) and 50 μm (right) and white arrows indicate cancerous cells invading into the lamina propria. In figures with bars and dots, all values reported as mean ± SEM. Statistical analyses, where shown, ^*^P < 0.05, ^**^P < 0.01, ^***^P < 0.001 by Student’s *t* test (**A**–**C**) and one-way ANOVA and Tukey test (**F**); each symbol represents a different mouse. In **E**, ^**^P < 0.01 by Chi-square comparing ND, LGD, and IMC between the two infected genotypes; ^§§§^*P* < 0.001 by Fisher’s exact test comparing cancer versus no cancer.

To determine the role of SMOX in gastric carcinogenesis during *H. pylori* infection, we first backcrossed C57BL/6 *Smox*^*–/–*^ mice [20] onto the FVB/N background until >99.5% of all tested alleles were FVB/N homozygous. Then, FVB/N INS-GAS mice, which are predisposed to develop dysplasia and intramucosal carcinoma (IMC) notably in response to *H. pylori* infection [21–23], were crossed to FVB/N *Smox*^*–/–*^ mice to obtain FVB/N INS-GAS *Smox*^*–/–*^ mice. In this report, wild-type (WT) and *Smox*^*–/–*^ mice refers to animals on the FVB/N INS-GAS background. We first confirmed that *Smox* mRNA expression was ablated in the gastric tissues of naïve *Smox*^*–/–*^ mice (Fig. 1A). In addition, *Smox*-deficient mice exhibited a significant reduction in the level of putrescine and spermidine in gastric tissues, but the spermine level was not affected (Fig. 1B); as expected, the spermidine to spermine ratio was significantly decreased in *Smox*^*–/–*^ mice compared to WT mice (Fig. 1B).

Then, mice were infected or not with *H. pylori* strain PMSS1 for 8 weeks. We first confirmed the induction of *Smox* in WT mice and knockdown of *Smox* mRNA in the *Smox*^*–/–*^ mice (Supplementary Fig. 1A) in the gastric tissues from *H. pylori*-infected animals. We found no difference in gastric colonization of *H. pylori* between the genotypes (Fig. 1C). As shown in the H&E staining, the gastric tissues of infected WT and *Smox*^*–/–*^ mice exhibited marked mucosal hyperplasia and infiltration of immune cells (Fig. 1D). In addition, areas of stromal reaction and neoplastic cells infiltrating the lamina propria, which are features of IMC, were observed in infected WT animals, whereas LGD, characterized by irregular and angulated glands, was the main finding in infected *Smox*^*–/–*^ mice (Fig. 1D). When the infiltration of immune cells was scored, we determined that gastritis severity was similar in both genotypes (Supplementary Fig. 1B). However, there was a striking, significant decrease in the development of IMC in mice lacking SMOX, with only 2 out of 29 infected *Smox*-deficient animals displaying cancer compared to 11 out of 22 cases in the infected WT animals (Fig. 1E). Additionally, the extent of dysplasia and carcinoma was significantly attenuated in infected *Smox*^*–/–*^ mice compared to WT mice (Fig. 1F). Furthermore, the infected *Smox*^*–/–*^ mice exhibited a significant loss of parietal cells, predominantly seen in the transitional mucosa of the junction of the antrum and corpus (Supplementary Fig. 2A), accompanied by a significant reduction in mucosa hyperplasia in the corpus (Supplementary Fig. 2B).

Note that in a 4-week infection model there were no differences in colonization, inflammation, nor cases of dysplasia between the infected WT and *Smox*^–/–^ mice (Supplementary Fig. 3A–C). However, we did observe a significant decrease in the extent of dysplasia in the infected *Smox*^–/–^ animals compared to controls (Supplementary Fig. 3D).

### SMOX-derived spermidine has no impact on carcinogenesis

Because *Smox*^*–/–*^ mice exhibited reduced gastric spermidine levels (Fig. 1B), we reasoned that SMOX may support cancer progression through the generation of spermidine. We thus supplemented infected mice with 14 mM spermidine in the drinking water, which corresponds to a 10-fold increase from the regular daily consumption [24]. In this new set of experiments, we again observed a significant reduction in the cases of cancer in the infected *Smox*^*–/–*^ mice compared to the infected WT animals. However, spermidine treatment did not have a significant effect on *H. pylori* colonization (Supplementary Fig. 4A) or inflammation (Supplementary Fig. 4B) in infected mice from either genotype. Strikingly, spermidine did not modify dysplasia or IMC in infected WT mice and did not restore progression to cancer in infected *Smox*^*–/–*^ mice (Supplementary Fig. 4C).

### SMOX sustains a procarcinogenetic transcriptome in infected stomachs

**Fig. 2:**
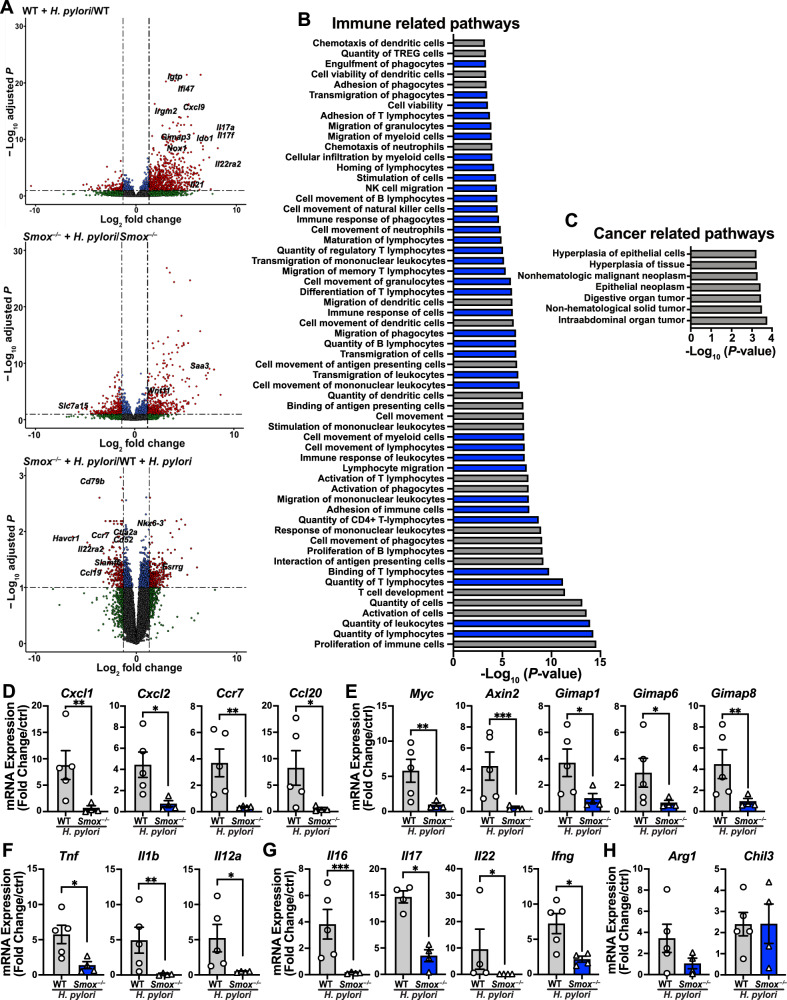
Transcriptional regulation orchestrated by SMOX in infected mice. **A** Transcriptomic analysis using RNA sequencing of infected and uninfected gastric tissues; *n* = 4–5 mice per genotype. The volcano plots show differential gene signatures in gastric tissues from infected mice compared with uninfected and infected mice (fold change >1.3; FDR < 0.05). Genes of interest bolded. **B**, **C** IPA was performed on the differential transcriptome dataset obtained from the gastric tissues from the infected WT and *Smox*^*–/–*^ mice and the pathways related to diseases and functions are broken down into immune (**B**) and cancer (**C**) related pathways. Blue bars, z-score < −2; gray bars, z-score < 0. Gene expression by RT-real-time PCR of genes encoding for chemokines (**D**), carcinogenic markers (**E**), proinflammatory cytokines (**F**), T cell cytokines (**G**), and anti-inflammatory markers (**H**). All values reported as mean ± SEM. Statistical analyses, where shown, ^*^P < 0.05, ^**^P < 0.01, ^***^P < 0.001 determined by one-way ANOVA and Tukey test or Kruskal–Wallis test followed by a Mann–Whitney *U* test.

To analyze the global molecular effect of SMOX in *H. pylori*-infected mice, we examined the gastric transcriptome of WT and *Smox*^–/–^ mice, infected or not with *H. pylori*, by RNA sequencing. Overall, we identified 27,284 sequences, comprising 20,943 known mRNAs and 6341 unknown sequences (Supplementary Dataset 1). For the analysis of the DEGs between groups, we focused on the genes upregulated or downregulated by 1.3–fold or more with *P* < 0.05 (Fig. 2A). In WT animals, there were 620 genes upregulated with *H. pylori* infection. These genes included those encoding for chemokines (*Cxcl1/2/3/5/9/10/11/13* and *Ccl19/20)*, for markers of the innate immune response (*Tnf, Il1a/b*, *Ido1*, *Acod1*, *Duoxa2*, *Nox1*), for autophagy and inflammation (*Irgm2, Igtp, Ifi47*), and for T-cell mediated responses (*Il16, Il17a, Il17f, Il21, Il22ra2, Ifng*; Fig. 2A). We also found an increase expression of *Gimap1/3/4/5/6/7/8/*9, genes which encode for GTPase IMAP (immunity-associated proteins) family members that have been implicated in cancer progression through the regulation of immune cell infiltration [25]. We found 396 and 183 genes increased and decreased, respectively, in *Smox*^–/–^ animals infected with *H. pylori* compared to uninfected controls; upregulated genes included those encoding for host response to bacterial infection (*Wnt11, Saa3*); downregulated genes included those involved in amino acid (*Slc7a15*, *Mme*) or nucleotide (*Tubal3*, *Cda*, *Mpped2*) metabolism, and regulation of transcription (*Tbx18*, *Fabp1*, *Fbp1*) and translation (*Hsd17b13*, *Rdh16f2*). When we compared the transcriptomic profiles of the two infected genotypes, 76 genes, including gastric cancer tumor suppressors involved in the Wnt/β-catenin signaling pathway (*Esrrg*, *Nkx6-3*), were upregulated by *Smox* deletion (Fig. 2A). Importantly, among the 113 genes downregulated in the infected *Smox*^–/–^ mice, we found the immune-related genes *Cd40/48/52/79b/83/86*, *Ctla2a*, *Ctla4*, *Slamf6*, *Sit1*, and *Havcr1*, and the procarcinogenic genes *Bcl2a1b/d*, *Pik3r6*, *Higd1b*, and *Gimap1/4/5/6/7/8*.

We then used Ingenuity Pathway Analysis (IPA) software on the significantly altered DEGs from the infected groups to determine the functional pathways regulated by SMOX at the transcriptional level (Supplementary Dataset 2). Most downregulated pathways in the infected *Smox*^–/–^ animals involved the immune system and cancer, such as “Migration of mononuclear leukocytes”, “Quantity of leukocytes”, “Immune response of cells” (Fig. 2B), and “Epithelial neoplasm” (Fig. 2C); the list of the genes corresponding to these pathways is provided in Supplementary Fig. 5A, B.

These alterations in immune and cancer related genes were further validated by targeted quantification of selected mRNAs in the tissues of WT and *Smox*^–/–^ animals. The chemokines *Cxcl1*, *Cxcl2*, *Ccr7*, and *Ccl20* (Fig. 2D); procarcinogenic genes *Myc*, *Axin2*, *Gimap1/6/8* (Fig. 2E); M1-like proinflammatory cytokines *Tnf*, *Il1b*, and *Il12a* (Fig. 2F); and T cell cytokines *Il16*, *Il17*, *Il22*, and *Ifng* (Fig. 2G) were significantly reduced in infected *Smox*^–/–^ animals compared to infected WT. There was no significant change in M2-like anti-inflammatory cytokines *Arg1* and *Chil3* (Fig. 2H). To further investigate the alterations in the immune cell infiltrate, we assessed the population of MPO-expressing cells by immunochemistry. Consistent with our RNA-sequencing and RT-real-time PCR results, we observed an increase in the infiltration of MPO^+^ cells in the infected WT animals that was significantly reduced in the infected *Smox*^–/–^ animals (Supplementary Fig. 6A, B).

### SMOX supports proliferation and DNA damage in GECs

**Fig. 3:**
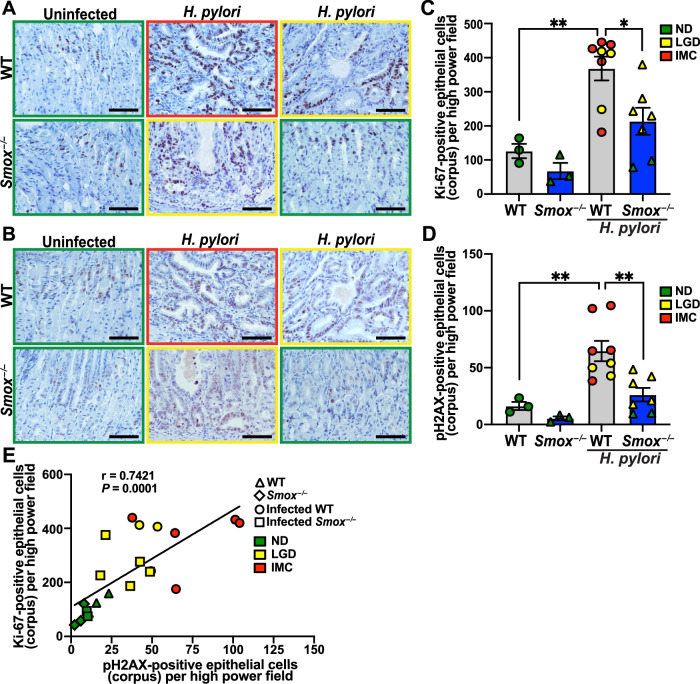
Cell proliferation and DNA damage in GECs. Representative images of gastric tissues immunostained for Ki-67 (**A**) and pH2AX (**B**); *n* = 3 uninfected mice and *n* = 7–8 infected mice per genotype; border and symbol color indicate stage of disease: ND, green; LGD, yellow; IMC, red. Scale bars, 50 μm. These stainings were used to quantify the number of Ki-67 (**C**) and pH2AX- (**D**) positive GECs from five high power fields per mouse. Each dot represents a mouse. Values are reported as mean ± SEM. Statistical analyses, where shown, ^*^P < 0.05, ^**^P < 0.01 determined by one-way ANOVA and a Tukey test. **E** Correlation between pH2AX and Ki-67 levels. Correlation and significance were determined by a simple linear regression.

Uncontrolled cell proliferation is a hallmark of cancer [26] and proliferating cells are more suspectable to DNA damage and tumorigenesis [27]. Accordingly, we observed an increase in Ki-67 (Fig. 3A) and pH2AX (Fig. 3B) nuclear immunostaining in GECs of infected WT animals compared to uninfected controls, as reliable markers of proliferation [28, 29] and DNA damage [20, 30], respectively. Both parameters were markedly reduced in the infected *Smox*^–/–^ mice (Fig. 3A, B). Quantification of the immunohistochemistry confirmed that *H. pylori* infection enhanced Ki-67 and pH2AX-positive cells in WT animals, which were significantly decreased in the infected *Smox*^–/–^ mice (Figs. 3C, D). Increased GEC DNA damage was significantly correlated with increased cell proliferation, with the most proliferation and DNA damage in the infected WT animals with low-grade dysplasia and cancer (Fig. 3E).

### SMOX-dependent acrolein generation by GECs during *H. pylori* infection

**Fig. 4:**
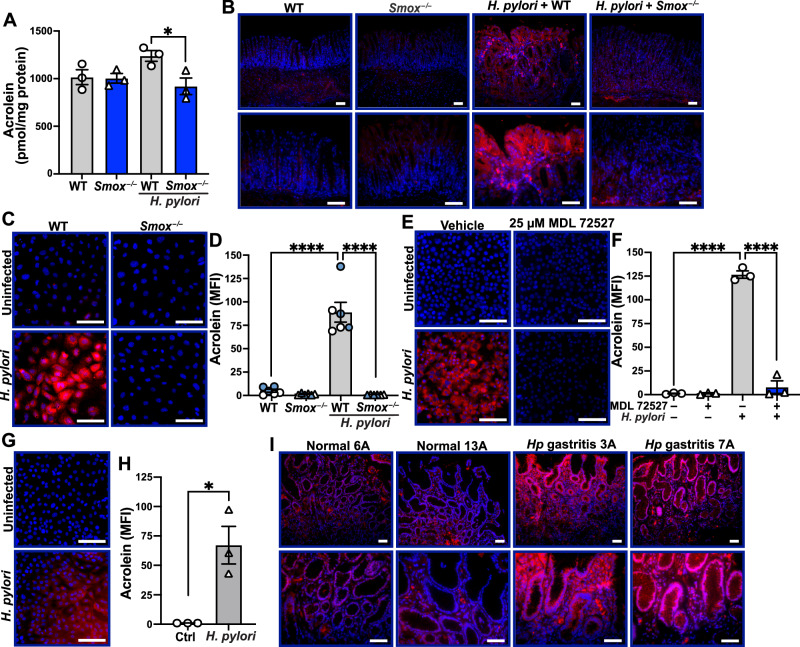
*H. pylori* induces SMOX-mediated acrolein production. **A** Acrolein quantification by mass spectrometry in the gastric tissues of experimental animals; *n* = 3 per genotype. **B** Representative immunofluorescence images of acrolein adducts from WT and *Smox*^*–/–*^ mice infected or not with *H. pylori*; data are representative of *n* = 2 uninfected mice and *n* = 3 infected mice per genotype. AcroleinRED assay was performed on gastric organoids generated from naïve mice and infected or not ex vivo for 24 h with *H. pylori* (**C**) and the staining was quantified (**D**); color of symbol indicates organoids derived from same mouse but different passages/experiments, with *n* = 2 animals per genotype and *n* = 3 replicates per mouse. AGS cells pretreated or not with the SMOX inhibitor MDL 72527 (25 μM) for 24 h followed by infection or not with *H. pylori* for 24 h were then stained with AcroleinRED (**E**) and the staining was quantified (**F**). Human gastric organoids isolated from normal patient biopsies infected or not with *H. pylori* were stained with AcroleinRED (**G**) and the staining was quantified (**H**). **I** The antral gastric tissues from 3 normal and 5 patients with *H. pylori* (*Hp*) gastritis were immunostained for acrolein adducts. In all representative fluorescence images, AcroleinRED or acrolein adducts are depicted in red and nuclei are stained in blue with DAPI. Scale bars, 50 μm (**B** and **I**, bottom row) or 100 μm (all other images). In all bar graphs, values are reported as mean ± SEM. Statistical analyses, where shown, ^*^P < 0.05, ^****^P < 0.0001 determined by one-way ANOVA and Tukey test (**A**, **D**, **F**) or Student’s *t* test (**H**).

To evaluate the role of acrolein in gastric carcinogenesis, we first measured the levels of free acrolein in mouse gastric tissues by mass spectrometry. Upon *H. pylori* infection, acrolein concentration was increased in WT mice and reduced in infected *Smox*^–/–^ mice (Fig. 4A). We then examined the level of acrolein-modified proteins in the gastric mucosa by immunofluorescence. There was an increase in acrolein adducts in WT mice infected with *H. pylori* compared to uninfected mice (Fig. 4B); the staining was observed in both GECs and immune cell infiltrates and was substantially reduced in infected *Sm*ox-deficient mice (Fig. 4B). Additionally, we generated gastric organoids from naïve WT and *Smox*^–/–^ mice, cultured them as monolayers, and then infected them with *H. pylori*. Then, we monitored acrolein synthesis using AcroleinRED, a live cell-based acrolein detection assay. Acrolein synthesis was induced in murine gastric organoids from WT mice when infected ex vivo with *H. pylori* but was greatly reduced in the infected *Smox*^–/–^ organoids (Fig. 4C). These results were confirmed by quantification of the fluorescence (Fig. 4D). Similarly, we observed that infection of the human GEC line AGS resulted in acrolein synthesis; this was markedly and significantly reduced in cells treated with the SMOX inhibitor MDL 72527 [9, 12] (Fig. 4E, F). We also found that *SMOX* mRNA expression was less expressed and acrolein was less synthesized in AGS cells infected with (1) the isogenic Δ*cagE* mutant compared to the parental strain PMSS1and (2) the *cagA*^–^ clinical isolate 3A compared to the *cagA*^+^ strains PMSS1 and 18C (Supplementary Fig. 7A–D), suggesting that CagA plays a role in SMOX-dependent production of acrolein by GECs. Next, we confirmed that patient-derived human gastric organoids also exhibited increased acrolein levels in response to *H. pylori* (Fig. 4G, H). Lastly, we determined that the level of acrolein protein adducts was increased in the gastric mucosa of endoscopic biopsies from patients with *H. pylori* gastritis and LGD compared to uninfected individuals (Fig. 4I and Supplementary Fig. 8). Notably, the staining was strongly abundant in GECs, but also present in the immune infiltrate.

### Scavenging acrolein dampens *H. pylori*-mediated carcinogenic events

**Fig. 5:**
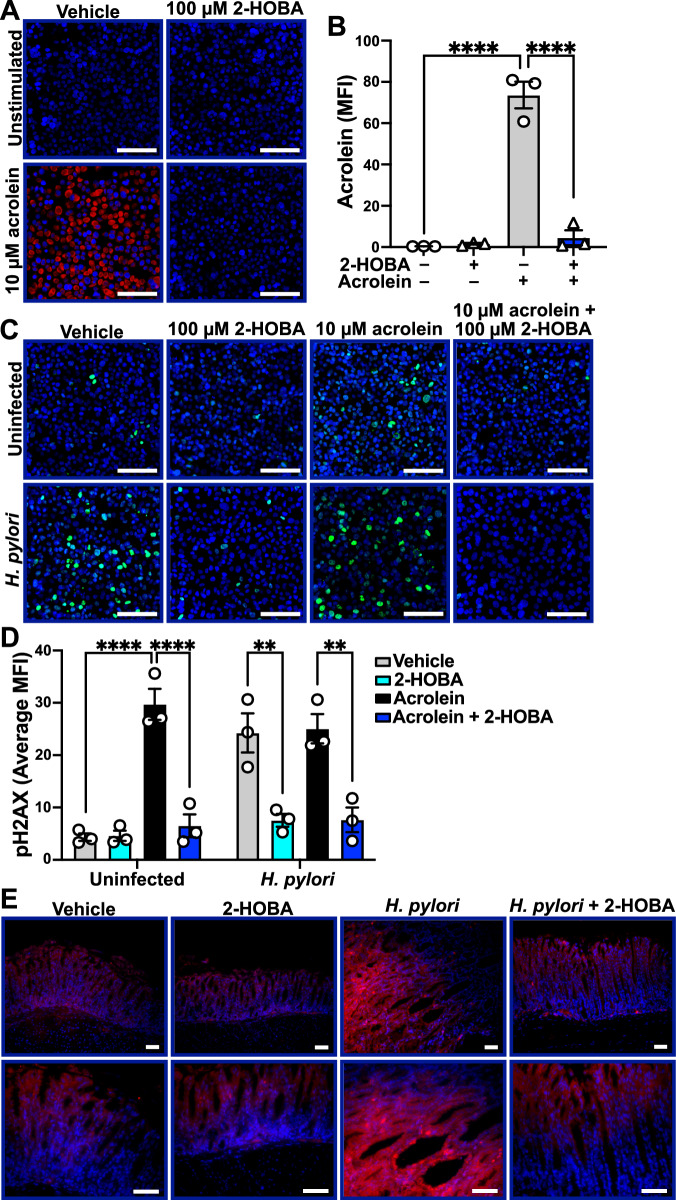
Effect of 2-HOBA on DNA damage. AGS cells pretreated or not with 2-HOBA (100 μM) for 2 h and then treated or not with acrolein (10 μM) for 24 h; cells were stained by the AcroleinRED assay (**A**), and the staining was quantified (**B**). **C**, **D** AGS cells were pretreated or not with 2-HOBA (100 μM) for 2 h and then exposed to acrolein (10 μM) and/or infected with *H. pylori*. After 24 h, cells were immunostained for pH2AX (green; **C**) and the fluorescence was quantified (**D**) from 3 independent experiments. **E** Representative immunofluorescence images of acrolein adducts in the gastric tissues of INS-GAS mice infected or not with *H. pylori* ± 2-HOBA; data are representative of *n* = 2 uninfected mice and *n* = 3 infected animals per group. Scale bars, 50 μm (**C** and **E**, bottom row) and 100 μm (all other images). Data represent the mean ± SEM. Statistical analyses, where shown, ^**^P < 0.01, ^****^P < 0.0001 by one-way ANOVA and Tukey test (**B**), and two-way ANOVA and Tukey test (**D**).

We recently reported that the electrophile scavenger 2-HOBA [19], which can react three times faster with electrophiles than lysine [24], prevents the development of dysplasia and carcinoma in FVB/N INS-GAS mice [22]. We thus questioned whether 2-HOBA can affect acrolein levels in infected GECs. First, we determined whether the electrophile scavenger 2-HOBA is able to scavenge acrolein in GECs. Pretreatment of AGS cells with 2-HOBA prevented the detection of free acrolein by AcroleinRED (Fig. 5A, B). Additionally, acrolein-induced DNA damage, determined by immunofluorescence for pH2AX, was markedly suppressed by 2-HOBA (Fig. 5C, D). pH2AX-staining induced by the exogenous acrolein addition was not further increased by *H. pylori* infection (Fig. 5C, D).

We next examined acrolein adducts in tissues from our prior study showing the protective effect of 2-HOBA in infected INS-GAS mice. Similar to Fig. 4B, gastric tissues from *H. pylori*-infected FVB/N INS-GAS mice exhibited more staining for acrolein adducts than uninfected animals (Fig. 5E), and this staining was strongly attenuated in the 2-HOBA-treated infected animals (Fig. 5E).

## Discussion

Current clinical strategies to eradicate gastric *H. pylori* include various combinations of antibiotics that are often ineffective due to the rise of antibiotic resistance [31]. Therefore, targetable alternative molecular pathways are needed to prevent disease progression to precancerous lesions and carcinoma in *H. pylori*-infected patients. In this study, we demonstrate that the genetic deletion of *Smox* prevents progression to cancer in a *H. pylori*-induced gastric cancer mouse model. Furthermore, we show a reduction in proinflammatory and procarcinogenic gene expression, along with a decrease in cell proliferation and DNA damage, in infected *Smox*^*–/–*^ animals. The generation of the highly reactive electrophile acrolein was induced during *H. pylori* infection in mouse and human GECs and reduced with *Smox* deletion or SMOX inhibition. Importantly, an electrophile scavenger, 2-HOBA, was able to attenuate acrolein levels and adducts, and acrolein-induced DNA damage, indicating the potential clinical relevance of this drug for infected patients.

We have previously shown that SMOX is involved in the host response to *H. pylori* infection, as there was a reduction in the infiltration of polymorphonuclear cells and a decrease in staining for myeloperoxidase expression in gastric tissues of infected C57BL/6 *Smox*^*–/–*^ mice compared to infected WT mice [20]. In the current study, although we observed no significant difference in the total number of leukocytes infiltrating into the gastric tissues between the two infected genotypes at 4 or 8 weeks, we did observe a difference in the function of the infiltrating cells. Notably, we found decreases in chemokines and innate and adaptive immune cell cytokines and a reduction in MPO-positive cells in the *Smox*^*–/–*^ mice infected with *H. pylori*. Since chronic inflammatory responses create an environment conducive to the development of cancer, we propose that it is the function of the cells rather the number present that is important in our model.

Previously our lab has implicated SMOX in mediating carcinogenic signaling in vitro. *Smox* deletion in mouse and human gastric organoids with genetic or chemical inhibition, respectively, exhibited a decrease in β-catenin activation during *H. pylori* infection [20]; notably *Myc* and *Axin2*, which are known β-catenin targets. In the present study, RNA sequencing data revealed that cancer pathways including “Epithelial neoplasm” and “Hyperplasia of epithelial cells” were downregulated in infected *Smox*^*–/–*^ mice. This was accompanied by a decrease in gene expression of cancer related genes (*Myc, Axin2, Gimap1/6/8*). Furthermore, we have shown that *H. pylori*-infected gerbils treated with the SMOX inhibitor MDL 72527 exhibited significantly reduced dysplasia and carcinoma [13], which was also seen in our genetic ablation of *Smox* in the FVB/N INS-GAS model. Taken together, these findings implicate SMOX as a key component in procarcinogenic signaling and promoting disease progression in gastric carcinogenesis during infection.

In the current report, we have identified acrolein, a byproduct of SMOX activity, as a critical inducer of DNA damage in GECs in response to *H. pylori* infection. Acrolein is a highly reactive aldehyde that can form covalent adducts with macromolecules such as DNA [32] and can then lead to genomic instability [14]. Mass spectrometry and immunofluorescence revealed an induction of free acrolein and acrolein-modified proteins, respectively, in *H. pylori*-infected WT animals, which was reduced by *Smox*^*–/–*^ deletion. Furthermore, *Smox*^*–/–*^ gastric organoids, along with human GECs and gastric organoids treated with the SMOX inhibitor MDL 72527 showed a reduction in acrolein generation during infection. Importantly, we also found that *H. pylori*-infected patients with gastritis or LGD exhibit more acrolein adducts in the gastric mucosa than uninfected patients, evidencing the clinical relevance in human disease. Supporting our finding, we have previously established that SMOX level increases with gastric histologic disease progression and that high SMOX expression correlates with increased oxidative DNA damage [13]. Furthermore, we previously reported that *SMOX* mRNA expression is CagA-dependent [12], similarly, here we show that CagA is needed for SMOX-dependent acrolein synthesis.

Given this, we contend that inhibition of SMOX activity and/or scavenging acrolein may prevent gastric cancer in *H. pylori*-infected patients. Recently, a series of potent and selective SMOX inhibitors have been identified and characterized to effectively inhibit SMOX activity [33]. However, additional testing examining pharmacokinetic factors and the translational feasibility of utilizing these inhibitors in vivo are required. We previously demonstrated that scavenging electrophiles attenuates the development of dysplasia and carcinoma in *H. pylori*-infected FVB/N INS-GAS mice [22, 23]; herein, we provide evidence for the first time that this compound can scavenge acrolein and reduce acrolein-mediated DNA damage. Since 2-HOBA is a natural product, safe and tolerable in healthy patients [34], and is being used in clinical trials for diseases such as rheumatoid arthritis (NCT05274243) and early Alzheimer’s disease (NCT06432166), this provides a rationale for its clinical use over other acrolein scavengers. Thus, our data have implicated SMOX-derived acrolein as a major contributing factor to gastric carcinogenesis and provides a further rationale for the use for 2-HOBA as a therapeutic intervention for gastric cancer prevention in high-risk patients.

## Materials and methods

### Ethics approval and consent to participate

All methods were performed in accordance with the relevant guidelines and regulations. Endoscopic gastric biopsies were obtained from patients at the Nashville Veterans Affairs Medical Center of the VA Tennessee Valley Healthcare System. Patients were undergoing esophagogastroduodenoscopy for clinically indicated reasons and provided informed consent for obtaining research biopsies under VA IRB protocol #1571167 (PI: Keith T. Wilson); clinical data was maintained in REDCap databases. Biopsies were also obtained from patients in Colombia [35]; for this study, individuals provided informed consent for obtaining the biopsies under the Institutional Review Board of Vanderbilt University IRB # 090460 and the Committee on Ethics of Universidad del Valle in Colombia Certificate # 023 of 2002.

The mice were used under protocols V2000018 and V2300022 approved by the Vanderbilt University Medical Center Institutional Animal Care and Use Committee and the Research and Development Committee of the Veterans Affairs Tennessee Valley Healthcare System. Procedures followed institutional policies, AAALAC guidelines, the AVMA Guidelines on Euthanasia, NIH regulations regarding the Guide for the Care and Use of Laboratory Animals, and the United States Animal Welfare Act of 1996.

### Human studies

Biopsies from the gastric antrum and corpus were fixed in 10% neutral buffered formalin, paraffin-embedded, and stained with hematoxylin and eosin (H&E). Each set of biopsies was scored by a gastrointestinal pathologist (MBP) for the presence or absence of chronic gastritis (mononuclear cells) and active gastritis (polymorphonuclear cells), and the microscopic detection of *H. pylori*. Tissues snap frozen in Brucella broth were subsequently homogenized and cultured for confirmation of the presence of *H. pylori* as in the mouse studies. Fresh tissues were also used for gastric organoid generation.

Human gastric tissues with low-grade dysplasia (LGD) were derived from adult individuals during a 16-year follow-up from a high cancer risk region in Colombia [35]. The diagnosis of LGD was the consensus reached by at least two out of three GI pathologists in the study.

### *H. pylori*

*H. pylori* PMSS1, a *cagA*^*+*^ strain with intact type IV secretion system function, was grown on Trypticase soy agar plates containing 10% sheep’s blood [20, 36]. For in vitro and in vivo infections, *H. pylori* was harvested from the plates and grown overnight in Brucella broth containing 10% fetal bovine serum (FBS). Then, for in vitro studies, the bacteria were resuspended in the media that the cells or organoids were growing in before infection. For infection, the overnight culture of *H. pylori* was diluted in fresh Brucella broth-FBS, grown, and then collected at the exponential phase.

*H. pylori* was isolated from endoscopic gastric biopsies from patients at the Nashville Veterans Affairs Medical Center of the VA Tennessee Valley Healthcare System. Briefly, biopsies were manually ground using a pestle in PBS, diluted, plated onto tryptic soy blood agar plates with antibiotics, and grown under anaerobic conditions. Single colonies were isolated and validated through Gram staining. In this study, we used the isolates 18C (*cagA*^+^) and 3A (*cagA*^*–*^) that were isolated from the corpus of patient 18 and the antrum from patient 3 in the VA study. Additionally, we utilized PMSS1^*ΔcagE*^, an isogenic mutant for the type IV secretion system that fails to translocate the cytotoxin-associated gene A (CagA) [12].

### Animals and infections

C57BL/6 WT and *Smox*^*–/–*^ mice [20, 37] were backcrossed to the FVB/N background and then crossed to transgenic FVB/N insulin-gastrin (INS-GAS) mice. These animals overexpress the human gastrin gene driven by a rat insulin I promoter. Mice were housed in a pathogen-free facility, with ventilated cage racks and were on a 12 h light-dark cycle. Male mice between 8 and 12 weeks were used for all studies. Only male mice were utilized since female mice are protected from *H. pylori*-induced gastric carcinogenesis [38, 39]. Mice were fed ad libitum with a defined rodent diet AIN-76A (BioServ) one week prior to the first infection and stayed on this diet until the experimental endpoint [21, 22]. Animals were infected by oral gavage with 10^9^ colony forming units (CFU) of *H. pylori* PMSS1 in 200 μL Brucella broth, two times, on days 0 and 2 [20, 21]. The control mice were gavaged with only broth on both days. In some studies, mice were treated or not with 14 mM spermidine (Sigma) in their drinking water, beginning two days after the second infection and the water was changed weekly [24]. 4 or 8 weeks after the first infection, mice were euthanized and stomachs were harvested as described [36, 40, 41]. Colonization was verified in all infected mice by counting the CFUs cultured after plating serial dilutions of homogenized gastric tissues [20, 36].

We also used gastric tissues from FVB/N INS-GAS animals infected or not with *H. pylori* PMSS1 ± 3 mg/mL 2-HOBA in the drinking water [22].

### Generation of mouse gastric organoids

Stomachs were dissected from WT and *Smox*^*–/–*^ animals at the gastroesophageal and gastroduodenal junctions. The forestomach was removed, and the stomachs were butterflied and washed thoroughly with PBS. Stomachs were incubated in 10 mM EDTA at 4 °C for 30 min on a rocker and then transferred to dissociation buffer (43.5 mM sucrose, 54.9 mM D-sorbitol). The tissue was vigorously shaken to release the glands. Gastric glands were plated in Matrigel on pre-warmed 24-well plates and maintained in Advanced DMEM/F12 supplemented with 50% conditioned media from L-WRN murine fibroblast cells [42] with penicillin-streptomycin, gentamicin, 10 μM Y-27632, and 10 μM SB431542. For two-dimensional monolayer culture, organoids were trypsinized and plated on poly-D-lysine and Matrigel coated 8-well chamber slides (Corning) overlaid with 50% conditioned media before infection with *H. pylori* PMSS1 at a multiplicity of infection (MOI) of 50.

### Generation of human gastric organoids

As described [43], organoids derived from adult human stomach biopsies were maintained and passaged. Gastric biopsies were obtained under IRB protocol # 1571167 as described above, and immediately placed in complete DMEM medium on ice in the endoscopy unit until gland isolation in the laboratory. Gastric glands were isolated and gastric epithelial monolayers were generated as described above for the mouse organoids. The epithelial monolayers were infected with *H. pylori* PMSS1 (MOI 50).

### AGS cells

AGS cells were obtained from ATCC, tested for mycoplasma contamination, and maintained in DMEM medium supplemented with 10% FBS, 10 mM HEPES, and 1% penicillin-streptomycin. AGS cells were treated with the SMOX inhibitor MDL 72527 (25 μM) for 24 h before infection, 2-HOBA (100 μM; TSI Co, Ltd.; Lot # SAA20200727) [22] for 2 h prior to stimulation, acrolein (10 μM, NSI Lab Solutions), and/or *H. pylori* (strains PMSS1, 18C, 3A, or PMSS1^*ΔcagE*^; MOI 100).

### Histopathology

A gastrointestinal pathologist (MBP) blinded to the experimental groups performed the histologic scoring using the modified Sydney System [44]. For each experimental mouse, a longitudinal strip of stomach tissue, including the corpus and antrum, was fixed in 10% neutral buffered formalin, paraffin-embedded, and stained with H&E. Acute and chronic inflammation in the corpus and antrum of the stomach were determined from 0–3 for each, leading to a final score of 0–12 [20, 36]. The presence and extent of LGD or IMC were determined from H&E sections [21–23]. On the mice infected for 8 weeks, the percent loss of parietal cells and extent of mucosa hyperplasia in the corpus on a 0–3 scale [28] was determined from H&E-stained sections.

### RNA analysis

RNA was isolated from a longitudinal strip of the mouse stomach, encompassing both the antrum and corpus, using the RNeasy Plus Mini Kit (QIAGEN). RNA was then sequenced as we described [36]. The Ovation RNA Sequencing System V2 (Tecan) was used for the generation and amplification of cDNA. RNASeq library preparation and Next Generation Sequencing (PE150) were performed using the NEBNext Ultra II Directional RNA Library Kit for Illumina (BioLabs, Inc) and Illumina NovaSeq6000 with NovaSeq 6000 SP Reagent Kit (Illumina), respectively. Reads were trimmed to remove the adaptor sequence and read quality was checked via *fastq*. The transcripts were quantified and mapped to the indexed mouse genome (M23, GRCm38) using *Salmon*. Transcript-level quantification was then summarized to the gene level, and then annotated and prepared for differential gene expression analysis using the R package *tximeta*. The R/Bioconductor package *DESeq2* was used to identify differentially expressed genes in each group using the Benjamini-Hochberg adjustment (FDR *P* < 0.05). The complete list of differentially expressed genes (DEGs), including Ensemble transcript identifiers, official gene symbols, fold changes, and *P* values, is provided in the Supplementary Dataset 1. We used Ingenuity Pathway Analysis software (IPA, QIAGEN) to determine disease functions that were significantly changed with infection, shown in Supplementary Dataset 2. A *P* value was calculated in IPA using Fisher’s exact test and then converted to a pathway score by converting the *P* value to the negative log of the *P* value for each pathway. The predicted activation state and the activation z-score was calculated based on the molecules within each pathway.

We also analyzed RNA expression by RT-real-time PCR. First, cDNA was synthesized using the SuperScript IV Reverse Transcriptase (Thermo Fisher) and Oligo d[T]_16_ Primer (Thermo Fisher). Then, Power SYBR Green PCR Master Mix (Thermo Fisher) and primers listed in Supplementary Table 1 were used to amplify cDNAs by RT-real-time PCR in the QuantStudio3 (ThermoFischer Scientific).

### Polyamine measurement in gastric tissues

Putrescine, spermidine, and spermine were quantified by liquid chromatography-mass spectrometry (LC-MS) using a Thermo TSQ Vantage Triple Quadrupole instrument operated in positive ion mode. As previously described [40], cell pellets were extracted using (70:30) acetonitrile/20 mM NH_4_OAc pH 8. Extracts were derivatized by reaction with 20 mM dansyl chloride in 100 mM NaHCO_3_ pH 10 for 20 min. The deuterated internal standards d_4_-pustrescine, d_8_-spermidine, and d_8_-spermine were used to quantify the polyamines [20, 28, 40].

### Measurement of acrolein

Acrolein levels were determined in the mouse stomach by LC-MS. Stomach tissues (10–20 mg) were homogenized in 10% acetonitrile (ACN), centrifuged at 8000 rpm for 10 min, and the supernatants were collected. A standard curve was generated by diluting a stock solution of 5 mg/ml acrolein (NSI Lab Solutions) in 90% methanol diluted in ACN. Danzyl hydrazone (10 mg/ml in ACN:10% acetic acid, 2:1; Sigma) was added to the samples and standards (200 μl), vortexed, and then incubated at room temperature for 1 h. The samples were quenched with 100 μl 500 mM glucose in 50% ACN and incubated for 30 min before being transferred into Eppendorf tubes for two extractions with 500 μl ethyl acetate. The top fraction was harvested, dried, and resuspended in 100 μl of 50% ACN. After centrifugation at 2000 rpm for 5 min, the supernatants were analyzed by LC-MS.

### Immunostaining

As previously reported [45], paraffin-embedded tissues were sectioned, heated for 1 h at 60 °C, and sections were deparaffinized in xylene and rehydrated in graded alcohols. For immunohistochemistry, endogenous peroxidase was blocked with 3% hydrogen peroxide solution for 30 min at room temperature. Antigen retrieval (Diva Decloaker, BioCare Medial) was performed for 20 min at 110 °C. Tissues were incubated with prediluted rabbit polyclonal anti-Ki-67 [28] (Biocare, Cat # PRM 325 AA), rabbit polyclonal anti-phospho-gamma H2AX [p Ser 139] polyclonal antibody [22] (pH2AX, 1:200, Novus, Cat # NB100-2280), or prediluted rabbit monoclonal anti-myeloperoxidase [28] (MPO, Biocare Cat # PP 023 AA) overnight at 4 °C. The secondary antibody, prediluted HRP Polymer anti-rabbit (DAKO), was applied for 1 h at room temperature. Visualization was performed using 3,3’-diaminobenzidine, and the tissues were counterstained by hematoxylin. The average number of Ki-67-positive and pH2AX-positive epithelial cells per 5 high powered fields in the corpus of the stomach and the average number of MPO-positive cells per 10 high powered fields in the corpus and antrum were quantified by our gastrointestinal pathologist (MBP) in a blinded manner.

For immunofluorescence on mouse and human tissues, paraffin-embedded tissues were sectioned, deparaffinized, and antigen retrieval was performed (DAKO Target Retrieval pH 9). Sections were treated with Proteinase K (DAKO) for 30 seconds, washed with PBS, incubated with 5% normal horse serum (Jackson ImmunoResearch 088-000-001) for 1 h at room temperature, and then incubated with Universal Protein Block (DAKO) for 1 h at room temperature. Tissues were incubated with monoclonal anti-acrolein adduct antibody (1:100, Thermo Fisher, Cat # MA5-27553) overnight at 4 °C. The secondary antibody, donkey anti-mouse IgG, Alexa Fluor 555 (1:600, Thermo Fisher, Cat # A-31570) was applied for 1 h at room temperature and then the slides were mounted with VECTASHIELD HardSet Antifade Mounting Medium with DAPI (Fisher Scientific). Images were taken on a Nikon Eclipse E800 microscope using SPOT 5.4 Software.

We also immunostained AGS cells cultured on chamber slides (LAB-TEK). After treatment or infection, cells were washed with PBS, fixed in 4% paraformaldehyde (ChemCruz) for 20 min at room temperature, washed with PBS, incubated with 100% cold methanol on ice for 5 min, washed with PBS, and blocked with Universal Protein Block (DAKO) for 40 min at room temperature. They were then incubated with the anti-phospho-gamma H2AX [p Ser 139] polyclonal antibody (1:200; Novus, Cat # NB100-2280) overnight at 4 °C followed by an incubation with Donkey anti-Rabbit IgG (H + L) Highly Cross-Adsorbed Secondary Antibody, Alexa Fluor Plus 488 (1:600; Thermo Fisher, Cat # A32790). Slides were mounted with VECTASHIELD HardSet Antifade Mounting Medium with DAPI (Fisher Scientific). Confocal images were acquired using a Cytation C10 Confocal Imaging Reader and Gen 5+ software (Aligent BioTek). Images were quantified using ImageJ.

### AcroleinRED assay

After stimulation with experimental conditions, human and mouse-derived gastric organoids and AGS cells plated on 8-well chamber slides were thoroughly washed with PBS. Each well was incubated with 10 μM AcroleinRED (DiagnoCine, Cat # FNK-FVD-0022) in DMEM for 30 min at 37 °C. All wells were thoroughly washed with PBS, fixed with 3.7% formaldehyde for 30 min at room temperature, washed with PBS, and mounted using the VECTASHIELD HardSet Antifade Mounting Medium with DAPI (Fisher Scientific). Slides were imaged using a Cytation C10 Confocal Imaging Reader and Gen 5+ software (Aligent BioTek). Images were quantified using ImageJ.

### Genotyping *Helicobacter pylori* for *cagA*

*H. pylori* was isolated from endoscopic gastric biopsies and grown from a single colony. Bacterial DNA was isolated using the DNeasy Kit for Blood and Tissue kit (Qiagen). The *cagA* and *16S* genes were amplified by RT-real-time PCR using PowerUp SYBR Green Master Mix (Thermo Fisher). Reactions were prepared with 10 ng of DNA and 500 nM primers. Sense and antisense primer sequences and PCR product sizes are as follows: *cagA*, 5′-GATAACAGGCAAGCTTTTGAGG-3′ and 5′ CTGCAAAAGATTGTTTGGCAGA-3′, 349 bp; *16S*, 5′-GGAGTACGGTCGCAAGATTAAA-3′ and 5′-CTAGCGGATTCTCTCAATGTCAA-3′, 127 bp. PCR products were run on a 3% agarose gel with 0.006% SybrSafe DNA Gel Stain (Invitrogen). Stained bands were visualized under UV light and photographed with a digital gel documentation system (Gel Doc EZ Imager and Image Lab Software version 5.1; Bio-Rad Labs Inc.).

### Statistics

Prism 10.2.1 (GraphPad Inc.) was used for statistical analysis and all results are expressed as mean ± SEM. At least 3 biological replicates were used for in vitro studies. Data that were not normally distributed according to the D’Agostino & Pearson normality test were log transformed. Outliers were identified using the ROUT test (Q = 5%) and removed from the analysis. Student’s *t* test was used to determine significant differences between two groups, whereas a one-way ANOVA followed by a Tukey test, or a Kruskal–Wallis and a Mann–Whitney *U* test was used for multiple groups. A two-way ANOVA followed by a Tukey test was used to determine significant differences between multiple groups on two different variables. Contingency analyses were performed by a Chi-square test or a Fisher’s exact test. For correlation analysis, simple linear regression was used to determine the r and *P* values.

## Supplementary information

Supplementary Figures

Dataset 1

Dataset 2

## Data Availability

The RNA sequencing data have been deposited in the NCBI Gene Expression Omnibus database under the accession number GSE266944.
