# Supplementary material for: Spermine oxidase promotes Helicobacter pylori-mediated gastric carcinogenesis through acrolein production
Source: Oncogene. 2024 Nov 10;44(5):296–306. doi: 10.1038/s41388-024-03218-7 (PMC11779639; doi:10.1038/s41388-024-03218-7)
Supplement: Supplementary file 1 — Supplementary Figures [file 41388_2024_3218_MOESM1_ESM.docx]

**Supplementary Fig. 1.** *Smox* expression and inflammation scores in WT and *Smox^–/–^* mice infected or not with *H. pylori* PMSS1 for 8 weeks. *Smox* mRNA expression by real-time RT-PCR from isolated RNA from gastric tissues infected or not with *H. pylori* (A); *n* = 6-7 uninfected mice and *n* = 12-14 infected mice per genotype. Inflammation scores from animals after an 8 week infection with *H. pylori* (B). (A, B) Each symbol represents a mouse, and the inflammation scores are the mean ± SEM of 3 independent experiments. Statistical analyses, where shown, ^*^*P* < 0.05, ^***^*P* < 0.001, ^****^*P* < 0.0001 determined by a one-way ANOVA followed by a Tukey test.

**Supplementary Fig. 2.** Effect of *Smox* deletion on *H. pylori* pathogenesis. Percentage of parietal cell loss (A) and mucosa hyperplasia (B) in the corpus of gastric tissues from WT and *Smox^–/–^* mice infected with *H. pylori* PMSS1 for 8 weeks; *n* = 21-29 infected mice per genotype; symbol color indicates stage of disease: ND, green; LGD, yellow; IMC, red. All values reported as mean ± SEM. Statistical analyses, where shown, ^*^*P* < 0.05 and ^***^*P* < 0.001 determined by a Student’s *t* test.

**Supplementary Fig. 3.** Colonization, inflammation, and carcinogenesis at 4 weeks post-infection. WT and *Smox^–/–^* mice were infected or not with *H. pylori* PMSS1 for 4 weeks. *H. pylori* colonization was determined by serial dilution and culture (A). Inflammation (B), frequency of dysplasia (C), and their extent (D) were determined from H&E-staining. All values reported as mean ± SEM. Statistical analyses, where shown, ^*^*P* < 0.05 determined by a one-way ANOVA followed by a Tukey test.

**Supplementary Fig. 4.** Spermidine supplementation does not reverse the phenotype in infected *Smox^–/–^* animals. WT and *Smox^–/–^* mice were infected or not with *H. pylori* PMSS1 and given 14 mM spermidine in the drinking water two days after the second infection. After 8 weeks, *H. pylori* colonization was determined by serial dilution and culture (A). Inflammation (B) and frequency of dysplasia and cancer (C) was determined from H&E-staining. (A, B) Each symbol represents a different mouse; data pooled from 2 independent experiments. All values reported as mean ± SEM. Statistical analyses, where shown, ^*^*P* < 0.05, ^**^*P* < 0.01, ^***^*P* < 0.001, ^****^*P* < 0.0001 determined by one-way ANOVA and Tukey test (B), and Chi-square test comparing the two infected genotypes with or without spermidine (C).

**Supplementary Fig. 5.** Genes and pathways altered in the infected *Smox^–/–^* mice in the RNA sequencing. Genes associated with the “Immune response of cells” pathway (A; associated with Fig. 2B) and “Epithelial neoplasm” pathway (B; associated with Fig. 2C). (A,B) Fold change > 1.3; FDR < 0.05.

**Supplementary Fig. 6.** Immunostaining for MPO. WT and *Smox^–/–^* INS-GAS mice were infected or not with *H. pylori* PMSS1 for 8 weeks. Representative images of gastric tissues immunostained for MPO (A) and quantification of the number of MPO^+^ cells per high power field (B); data are representative of *n* = 5 uninfected mice and *n* = 10 infected mice per genotype. Scale bars, 50 μm. All values reported as mean ± SEM; ^**^*P* < 0.01 and ^***^*P* < 0.001 determined by a one-way ANOVA followed by a Tukey test.

**Supplementary Fig. 7.** CagA is required for *SMOX* expression and acrolein production by GECs. The *cagA* status of the strains was assessed by PCR (A). AGS cells were infected with *H. pylori* PMSS1, PMSS1*^ΔcagE^*, 18C, or 3A. *SMOX* mRNA expression was determined by RT- real-time PCR after 6 h (B). The AcroleinRED assay was performed after 24 h (C) and the staining was quantified (D); acrolein adducts are depicted in red and nuclei are stained in blue with DAPI. Scale bars, 100 μm. All values are reported as mean ± SEM. Statistical analyses, where shown, ^*^*P* < 0.05, ^**^*P* < 0.01, ^****^*P* < 0.0001 determined by one-way ANOVA and Tukey test (B, D). Data are derived from 3 independent experiments.

**Supplementary Fig. 8.** Acrolein production is increased in *H. pylori*-infected patients with LGD. The antral gastric tissues from 3 normal patients and 8 *H. pylori*-infected patients with LGD were immunostained for acrolein adducts. Representative images of acrolein adducts in normal and LGD samples (top row) with H&E-staining of serial sections from the same tissues to depict the normal or dysplasia glands, respectively (bottom row). Acrolein adducts are depicted in red and nuclei are stained in blue with DAPI. Scale bars, 50 μm.

**Supplementary Table 1.** List of RT-PCR primers used in this paper (m, mouse; h, human).

| **Target** | **Sequence** |
| --- | --- |
| *mSmox* | F: CACGTGATTGTGACCGTTTC |
|  | R: TCATCCTCCCACACGAACTG |
| *hSMOX* | F: GCGGCTTTGATGTCCTCTAC |
|  | R: CACTGCCTCGTCATCACACT |
| *Cxcl1* | F: GCTGGGATTCACCTCAAGAA |
|  | R: CTTGGGGACACCTTTTAGCA |
| *Cxcl2* | F: GCCAAGGGTTGACTTCA |
|  | R: TGTCTGGGCGCAGTG |
| *Ccr7* | F: CAGGTGTGCTTCTGCCAAGAT |
|  | R: GGTAGGTATCCGTCATGGTCT |
| *Ccl20* | F: CGACTGTTGCCTCTCGTACA |
|  | R: AGGAGGTTCACAGCCCTTTT |
| *Myc* | F: ACGACAAGAGGCGGACACA |
|  | R: GATGTAGGCGGTGGCTTTTTT |
| *Axin2* | F: CCAGTCAATCCTTATCACGTAGGTT |
|  | R: CAGTGCGTCGCTGGATAACTC |
| *Gimap1* | F: ATGGTTGCGTGCTTGGTGA |
|  | R: CTCAGCCACTATCTCTGCCA |
| *Gimap6* | F: TGGCTTTACAGAAAAACACTCGG |
|  | R: TGGGGTCGAAATGTAGATTCTCT |
| *Gimap8* | F: ACCAGGGAACGTCCACACT |
|  | R: GCATCTGTCGGTTACCATGTGA |
| *Tnf* | F: CTGTGAAGGGAATGGGTGTT |
|  | R: GGTCACTGTCCCAGCATCTT |
| *Il1b* | F: ACCTGCTGGTGTGTGACGTTCC |
|  | R: GGGTCCGACAGCACGAGGCT |
| *Il12a* | F: AAATGAAGCTCTGCATCCTGC |
|  | R: TCACCCTGTTGATGGTCACG |
| *Arg1* | F: AAGAAAAGGCCGATTCACCT |
|  | R: CACCTCCTCTGCTGTCTTCC |
| *Chil3* | F: ACTTTGATGGCCTCAACCTG |
|  | R: AATGATTCCTGCTCCTGTGG |
| *Il16* | F: AAGAGCCGGAAATCCACGAAA |
|  | R: GTGCGAGGTCTGGGATATTGC |
| *Il17* | F: ATCCCTCAAAGCTCAGCGTGTC |
|  | R: GGGTCTTCATTGCGGTGGAGAG |
| *Il22* | F: TTGAGGTGTCCAACTTCCAGCA |
|  | R: AGCCGGACGTCTGTGTTGTTA |
| *Ifng* | F: GGCCATCAGCAACAACATAAGCGT |
|  | R: TGGGTTGTTGACCTCAAACTTGGC |
| *Actin* | F: CCAGAGCAAGAGAGGTATCC |
|  | R: CTGTGGTGGTGAAGCTGTAG |

**Supplementary Datasets**

**Dataset 1.** The complete list of differentially expressed genes (DEGs), including Ensemble transcript identifiers, official gene symbols, fold changes, and *P* values for all four groups (*n* = 4-5 per genotype).

**Dataset 2.** Full list of pathways identified by IPA performed on the DEG dataset between uninfected and infected groups presented in Dataset 1.
